# Antiparasitic Properties of Cardiovascular Agents against Human Intravascular Parasite *Schistosoma mansoni*

**DOI:** 10.3390/ph14070686

**Published:** 2021-07-16

**Authors:** Raquel Porto, Ana C. Mengarda, Rayssa A. Cajas, Maria C. Salvadori, Fernanda S. Teixeira, Daniel D. R. Arcanjo, Abolghasem Siyadatpanah, Maria de Lourdes Pereira, Polrat Wilairatana, Josué de Moraes

**Affiliations:** 1 Research Center for Neglected Diseases, Guarulhos University, Praça Tereza Cristina 229, São Paulo 07023-070, SP, Brazil; raquel.porto@sereducacional.com (R.P.); ana.npdn@gmail.com (A.C.M.); ragabi2009@gmail.com (R.A.C.); 2 Institute of Physics, University of São Paulo, São Paulo 05508-060, SP, Brazil; mcsalva@if.usp.br (M.C.S.); nandast@if.usp.br (F.S.T.); 3 Department of Biophysics and Physiology, Federal University of Piaui, Teresina 64049-550, PI, Brazil; daniel.arcanjo@ufpi.edu.br; 4 Ferdows School of Paramedical and Health, Birjand University of Medical Sciences, Birjand 9717853577, Iran; asiyadatpanah@yahoo.com; 5 CICECO-Aveiro Institute of Materials & Department of Medical Sciences, University of Aveiro, 3810-193 Aveiro, Portugal; mlourdespereira@ua.pt; 6 Department of Clinical Tropical Medicine, Faculty of Tropical Medicine, Mahidol University, Bangkok 10400, Thailand

**Keywords:** antiparasitic, anthelmintic, antischistosomal, drug repurposing, amiodarone, cardiovascular agents, antiarrhythmic, schistosomiasis

## Abstract

The intravascular parasitic worm *Schistosoma mansoni* is a causative agent of schistosomiasis, a disease of great global public health significance. Praziquantel is the only drug available to treat schistosomiasis and there is an urgent demand for new anthelmintic agents. Adopting a phenotypic drug screening strategy, here, we evaluated the antiparasitic properties of 46 commercially available cardiovascular drugs against *S. mansoni.* From these screenings, we found that amiodarone, telmisartan, propafenone, methyldopa, and doxazosin affected the viability of schistosomes in vitro, with effective concentrations of 50% (EC_50_) and 90% (EC_90_) values ranging from 8 to 50 µM. These results were further supported by scanning electron microscopy analysis. Subsequently, the most effective drug (amiodarone) was further tested in a murine model of schistosomiasis for both early and chronic *S. mansoni* infections using a single oral dose of 400 mg/kg or 100 mg/kg daily for five consecutive days. Amiodarone had a low efficacy in chronic infection, with the worm and egg burden reduction ranging from 10 to 30%. In contrast, amiodarone caused a significant reduction in worm and egg burden in early infection (>50%). Comparatively, treatment with amiodarone is more effective in early infection than praziquantel, demonstrating the potential role of this cardiovascular drug as an antischistosomal agent.

## 1. Introduction

Schistosomiasis, also known as snail fever and bilharzia, is a parasitic disease caused by infection with an intravascular trematode of the genus *Schistosoma* [1]. The disease infects approximately 230 million people, with more than 750 million at risk of infection, and results in significant mortality and devastating social and economic consequences [2]. Disease morbidity, due to inflammation and fibrosis associated with eggs laid by the adult worms, can be painful and debilitating, hampering both personal productivity and community development. In children, schistosomiasis can cause anemia, malnutrition, growth stunting, intellectual retardation, and cognitive deficits [3]. *Schistosoma mansoni* is the species prevalent in Africa, the Middle East, South America, and the Caribbean, especially in poor and rural communities without access to safe drinking water and adequate sanitation [2].

Although schistosomiasis is globally massive in its impact, only one drug is currently available, namely, praziquantel, and it has been used for decades as the key agent for treating infections caused by schistosomes [4]. Despite its effectiveness, the heavy reliance on a single drug bears the risk of drug resistance development. The reduced efficacy of praziquantel after multiple rounds of mass drug administration has also been well documented [5]. Additionally, praziquantel is well known to be more effective against adult (chronic infection) than juvenile parasites (early infection), a property that likely contributes to the failure of praziquantel to cure schistosome infections completely [6]. Therefore, the search for new efficient antischistosomal compounds is crucial.

As a disease of poverty, also known as a neglected disease, schistosomiasis sufferers will never be viewed as a viable target market for the pharmaceutical industry [7]. In this context, finding new indications for existing drugs, also known as drug repositioning or reprofiling, may be considered a promising strategy to accelerate drug discovery and development in combating schistosomiasis [8,9]. In recent years, using an in vitro phenotypic screening assay against *S. mansoni* adult worms, we have screened many clinical drugs that have been approved by either the US Food and Drug Administration or its foreign equivalents, and identified several promising compounds [10,11,12,13,14,15]. Among these, from a screening of 13 diuretics, we identified that spironolactone, a potassium-sparing diuretic, had potent antischistosomal effects in vitro and in vivo in a mouse model of schistosomiasis [15].

The aim of this study was to evaluate the antischistosomal properties of commercially available cardiovascular agents, except diuretics, against blood fluke *S. mansoni*. Adopting a phenotypic drug screening strategy, 46 drugs were first tested in vitro against adult parasites ex vivo and, subsequently, the effective concentrations of 50% (EC_50_) and 90% (EC_90_) values against schistosomes were determined. The motility and morphology of the schistosomes were also monitored using light and scanning electron microscopy. Finally, the most effective drug (amiodarone, a potassium channel blocker antiarrhythmic) was tested in vivo using either an early or a chronic *S. mansoni* murine model to characterize the full spectrum of activity of this drug.

## 2. Results

### 2.1. Phenotypic Screening of Cardiovascular Agents against Schistosomes

**Figure 1:**
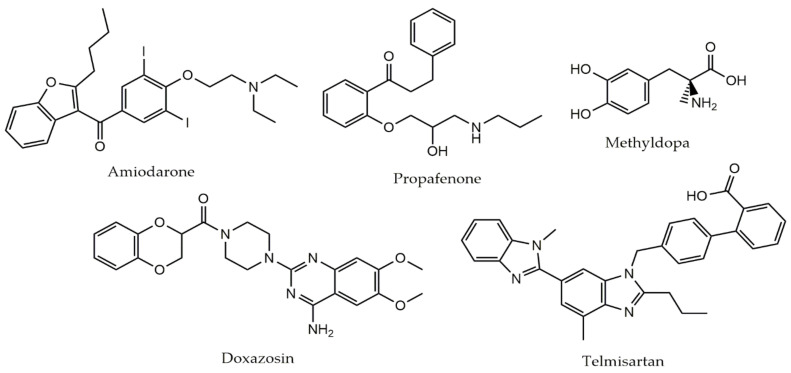
Chemical structures of cardiovascular drugs with in vitro schistosomicidal activity.

The 46 cardiovascular drugs were initially screened (primary screening) against adult schistosomes ex vivo at 50 µM. Of all the drugs tested, five (amiodarone, doxazosin, methyldopa, propafenone, and telmisartan) showed antiparasitic activity after 72 h, and these compounds were further tested (secondary screening) at a range of concentrations for their EC_50_ and EC_90_ determination, aiming to discriminate the most potent compound. Results of the EC_50_ and EC_90_ value for each tested drug, separated by class, are summarized in Table 1. The gold-standard antiparasitic compound praziquantel was used as a positive control. The chemical structures of these five drugs selected in primary screening are shown in Figure 1.

Among the five cardiovascular agents selected, amiodarone was the most active drug, with EC_50_ and EC_90_ of 8.2 and 11.4, respectively. Telmisartan and propafenone also displayed high schistosomicidal activity (EC_50_ < 25 μM), whereas methyldopa and doxazosin exhibited moderate antischistosomal properties (EC_50_ > 30 μM). Using the log rank (Mantel–Cox) test, comparison of schistosomicidal activity revealed that the order of potency was amiodarone (*p* < 0.01), telmisartan (*p* < 0.001), propafenone (*p* < 0.001), methyldopa (*p* < 0.01), and doxazosin (*p* < 0.001). For the remaining compounds tested, no EC_50_ and EC_90_ could be calculated, due to lack of activity at 50 μM. For comparison, the EC_50_ value of praziquantel was of 0.98 μM.

### 2.2. Dose-Dependent Effects of Cardiovascular Agents against Schistosomes

**Figure 2:**
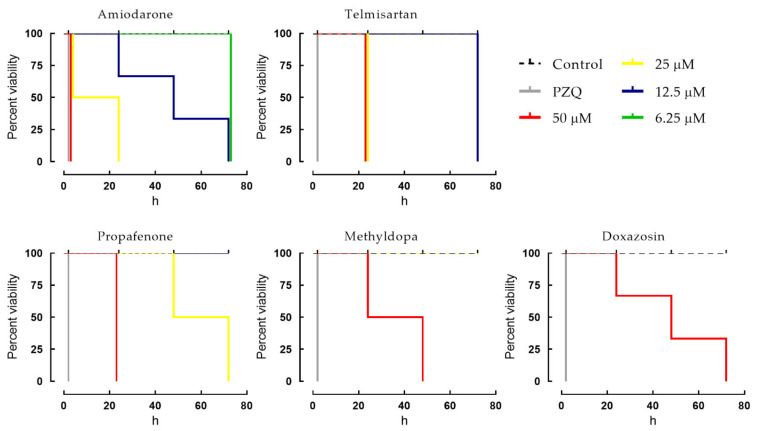
Viability of *S. mansoni* ex vivo following exposure to cardiovascular agents. Adult worms were obtained from mice by perfusion 49 days after infection. Parasites were monitored for up to 72 h and survival was plotted as a percentage over time using the Kaplan–Meier curves. Mean values were derived from a minimum of three experiments, and each experiment was performed with five replicates. Control (dashed line): RPMI 1640 + 0.5% DMSO. PZQ, praziquantel at 2 μM.

To further evaluate the efficacy of the five cardiovascular agents that exhibited in vitro schistosomicidal activity, we investigated the temporal effects of different concentrations of drugs on *S. mansoni*. In this case, the Kaplan–Meier survival curves were performed to explore the influence of these drugs on the overall survival of parasites (Figure 2). The gold-standard anthelmintic drug praziquantel was used as a positive control. Control parasites remained viable over the entire observation period of 72 h. However, it was observed that the five cardiovascular drugs induced mortality in adult parasites in a time- and concentration-dependent manner when compared to the negative control. In more detail, at a concentration of 50 μM, amiodarone, telmisartan, and propafenone had a lethal effect on all schistosomes within 24 h of contact. At 25 μM, only amiodarone and telmisartan exhibited schistosomicidal activity against all parasites within 24 h. A slightly slower onset of action was recorded when parasites were exposed to methyldopa and doxazosin. In contrast, praziquantel displayed a fast onset of action against all adult parasites, and this drug produced instantaneous contraction of the muscles of the parasite, followed by spastic paralysis of the schistosomes.

### 2.3. Effects of Cardiovascular Agents on the Tegument of Schistosomes

**Figure 3:**
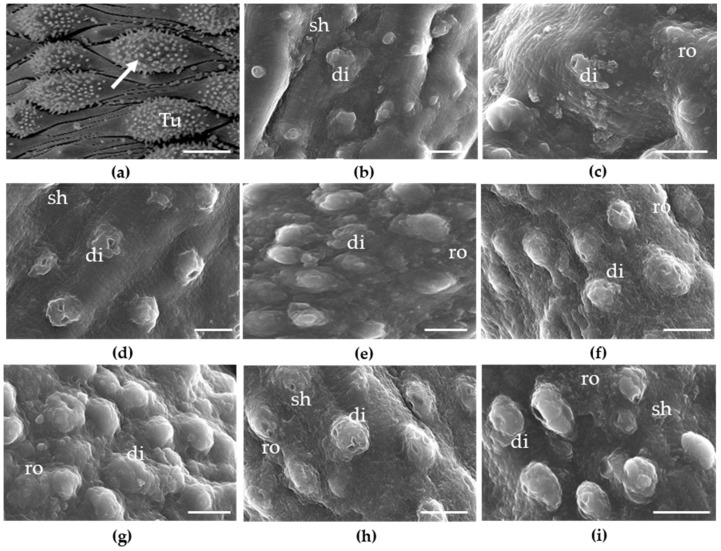
Microscopy observations of *S. mansoni* male worms after exposure to cardiovascular drugs. Freshly perfused parasites were placed on plates containing various concentrations of cardiovascular agents. (**a**) Control showing tubercles (Tu) and spines on the surface (arrow); (**b**) amiodarone 12.5 µM; (**c**) amiodarone 25 µM; (**d**) amiodarone 50 µM; (**e**) telmisartan 12.5 µM; (**f**) telmisartan 25 µM; (**g**) telmisartan 50 µM; (**h**) propafenone 50 µM; (**i**) propafenone 25 µM. Parasites were monitored for up to 72 h and micrographs of the mid-body region of schistosomes show disintegration (di), roughening (ro), and shrinking (sh). Images were obtained using a JEOL SM-6460LV scanning electron microscope. Scale-bars: 10 μm.

To determine whether cardiovascular drugs cause morphological alterations to the *S. mansoni* tegument, we performed scanning electron microscopy analysis, prioritizing the three most potent compounds, i.e., amiodarone, telmisartan, and propafenone. Results of the microscopy examination are shown in Figure 3. Microscopic images revealed that the dorsal surface of *S. mansoni* male worms incubated in drug-free medium (control group) had an intact tegument, bearing large numerous tubercles with evenly distributed spines. However, compared with the control groups, parasites exposed to any of the three cardiovascular agents displayed substantial tegumental disruption throughout the body. For example, the tubercles of male worms demonstrated shrinkage, and the spicules were markedly affected. Roughening and disintegration of the schistosomes’ surface were also observed after exposure to amiodarone, telmisartan, or propafenone.

### 2.4. Antischistosomal Properties of Amiodarone in Mice Harboring Either Chronic or Early S. mansoni Infections

In the final stage of the study, we evaluated the most potent cardiovascular drug (amiodarone, EC_50_ < 10 µM) in mice harboring either early or chronic *S. mansoni* infections. In both stages of infections, amiodarone was administered using a single oral dose (400 mg/kg) or once daily for five consecutive days (100 mg/kg/day). It should be noted that these doses were selected because they are often used in studies on approaches to drug discovery for schistosomiasis [10,11,12,13,14,15]. For comparison, data obtained with the drug of reference (praziquantel at 400 mg/kg) in animals with either chronic or early infections are also presented. In all treatments, parasites were quantified after perfusion of mice, and egg development stages (oogram) and fecal egg load were determined. Results were compared with the infected but untreated control animals.

#### 2.4.1. Effect of Amiodarone on Worm Burden

**Figure 4:**
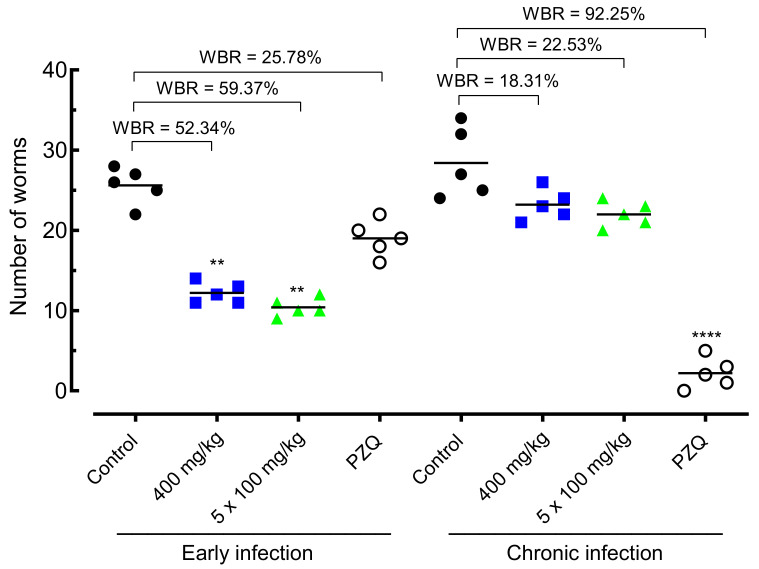
Effect of amiodarone on the parasite burden of mice harboring either early or chronic *S. mansoni* infection. Amiodarone (single dose of 400 mg/kg or 100 mg/kg for five consecutive days), praziquantel (PZQ, 400 mg/kg), and vehicle (control) were administered 21 days (early infection) or 49 days (chronic infection) post-infection by oral gavage. On day 63 post-infection, all animals were euthanized, and parasite burdens were determined. Points represent data from individual animals (*n* = 5 per group). Horizontal bars represent median values. ** *p* < 0.01, **** *p* < 0.0001 compared with infected untreated control. WBR, worm burden reduction.

The effect of amiodarone on the worm burden of *S. mansoni*-infected animals is shown in Figure 4. In early infection, administration of amiodarone at a single dose of 400 mg/kg or 100 mg/kg daily for five days resulted in significant worm burden reductions of 52.34% (*p* < 0.01) and 59.37% (*p* < 0.01), respectively, compared to *S. mansoni*-infected control-group mice. Praziquantel achieved low, and statically nonsignificant, worm burden reductions of 25.78% when animals with early infections were orally treated at a dose of 400 mg/kg.

In chronic *S. mansoni* infection, amiodarone resulted in low worm burden reductions of 18–23% in both drug regimens (single dose of 400 mg/kg or 100 mg/kg daily). This reduction in the number of parasites was statically insignificant. In contrast, praziquantel at 400 mg/kg achieved high total worm burden reductions of 92.25% (*p* < 0.0001).

#### 2.4.2. Effect of Amiodarone on Egg Burden

**Figure 5:**
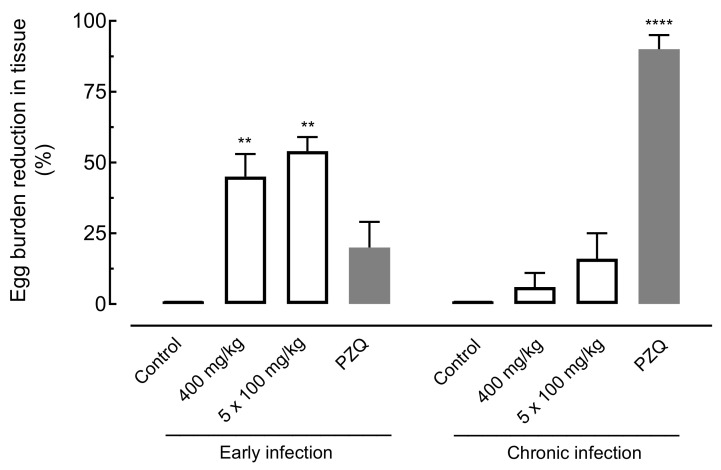
Effect of amiodarone on the egg burden in the tissue of mice harboring either early or chronic *S. mansoni* infection. Amiodarone (single dose of 400 mg/kg or 100 mg/kg for five consecutive days), praziquantel (PZQ, 400 mg/kg), and vehicle (control) were administered 21 days (early infection) or 49 days (chronic infection) post-infection by oral gavage. On day 63 post-infection, all animals were euthanized, and egg burdens were determined by counting immature eggs in the intestine. Points represent data from individual animals (*n* = 5 per group). Horizontal bars represent median values. ** *p* < 0.01, **** *p* < 0.0001 compared with infected untreated control.

**Figure 6:**
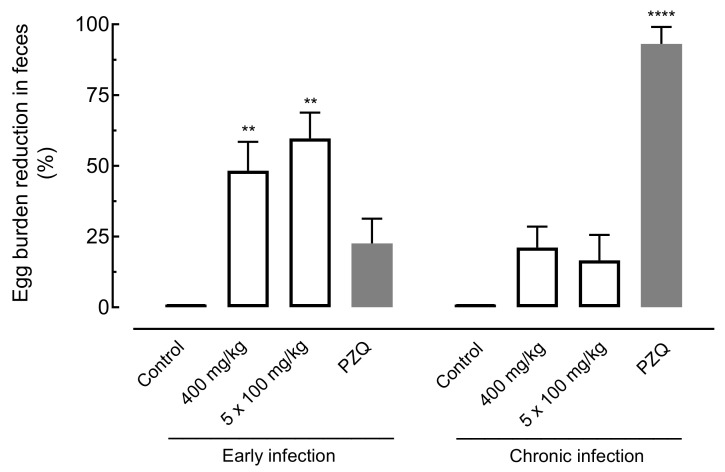
Effect of amiodarone on the egg burden in the tissue of mice harboring either early or chronic *S. mansoni* infection. Amiodarone (single dose of 400 mg/kg or 100 mg/kg for five consecutive days), praziquantel (PZQ, 400 mg/kg), and vehicle (control) were administered 21 days (early infection) or 49 days (chronic infection) post-infection by oral gavage. On day 63 post-infection, all animals were euthanized, and egg burdens were determined by counting immature eggs in the intestine. Points represent data from individual animals (*n* = 5 per group). Horizontal bars represent median values. ** *p* < 0.01, **** *p* < 0.0001 compared with infected untreated control.

The effect of amiodarone on the egg burden of *S. mansoni*-infected mice was evaluated using the oogram technique (eggs in the intestine) and the Kato–Katz technique (eggs per gram in feces). Results are summarized in Figure 5 and Figure 6.

Regarding eggs in the intestine, oral treatment with amiodarone at a single dose of 400 mg/kg or 100 mg/kg daily for five days in mice harboring early infections significantly reduced the number of immature eggs by 45.82% (*p* < 0.01) and 54.26% (*p* < 0.01), respectively. In animals with chronic *S. mansoni* infection, low worm burden reductions of 6.23% and 16.81% were observed for amiodarone, whereas praziquantel resulted in an egg burden reduction of 90.08% (Figure 5).

With respect to fecal examination, the use of amiodarone at a single dose (400 mg/kg) or daily for five days (100 mg/kg) presented significant decreases in egg burden of 48.31% (*p* < 0.01) and 59.5% (*p* < 0.01), respectively, in mice with early infection. In the same stage of infection, lower egg burden reduction values were obtained for praziquantel (22.67%). In chronic infection, amiodarone showed a nonsignificant reduction in the number of eggs when compared to infected control-group mice. In contrast, the number of eggs was highly reduced when praziquantel was administered to animals with chronic *S. mansoni* infection (Figure 6).

## 3. Discussion

In this study, we examined the influence of various commercially available cardiovascular drugs on the viability of human blood fluke *S. mansoni*. Using a phenotypic screening assay, we demonstrated that amiodarone, telmisartan, propafenone, methyldopa, and doxazosin affected the viability of adult schistosomes in vitro. These drugs were further tested (secondary screening), aiming to discriminate the most potent compound for a sustainable animal model trial. Finally, we demonstrated that antiarrhythmic drug amiodarone exhibits anthelmintic properties in a murine model of schistosomiasis, and this in vivo effect was associated with significant reductions in worm burdens and egg production, specifically for early *S. mansoni* infections.

Our decision to investigate cardiovascular agents as a possible therapeutic axis for schistosomiasis was triggered by our previous phenotypic (whole-organism) screening of a small-molecule collection that included drugs approved for use with humans [10,12,15]. Specifically, cardiovascular agents emerged as one of several drug classes that consistently affected the viability of adult schistosomes after 72 h at ~10 µM. For example, from a screening of 13 diuretics, spironolactone, a potassium-sparing diuretic, had potent antischistosomal effects on adult schistosomes in vitro and in a murine model of schistosomiasis [15]. In vitro, spironolactone at low concentrations (EC_50_ of 7.2 µM) was able to alter worm motor activity and the morphology of adult schistosomes. In vivo, oral treatment with spironolactone at a single dose (400 mg/kg) or daily for five consecutive days (100 mg/kg/day) in mice harboring either early or chronic *S. mansoni* infections significantly reduced worm burden and egg production. Thus, our present data are consistent with and expand upon those from these findings and encourage further exploration of cardiovascular agents to search for new antischistosomal agents. In terms of the methodologies employed, the in vitro phenotypic screening, route of administration (oral), dosing regimen applied (400 mg/kg or 100 mg/kg/day), and model of *S. mansoni* infection (early and chronic) are in accordance with various studies for antischistosomal drug discovery [10,11,12,13,14,15].

In vitro, the effects of cardiovascular drugs on parasite viability were quantified and analyzed over many time points, replicates, and trials. All five drugs showed clear dose-dependent effects. However, not all drugs had the same potency. Amiodarone had the most potent antischistosomal activity, with an EC_50_ of ~8 μM. A similar potency and efficacy were observed for telmisartan (EC_50_ of ~12 μM), but the other cardiovascular drugs had markedly less potency. Comparatively, although amiodarone was less potent than praziquantel, few other studies have described compounds with schistosomicidal activity at a concentration below 10 μM (for review, see [9,16]), which highlights the importance of amiodarone as an anthelmintic agent. Indeed, the 10 µM concentration defines a cut-off value for the selection of drugs for further exploration in an animal model, whereas the 50 µM screening was included to avoid the loss of chemical information for exploring the structure–activity relationship and/or medicinal chemical optimization [12,17].

Given the importance of the schistosomes’ tegument as a target for drugs [18,19,20], we used scanning electron microcopy to examine the surface of worms exposed to amiodarone, telmisartan, and propafenone. We found that treatment with any of the three cardiovascular drugs significantly damaged the worm’s tegument, usually with disintegration of tubercles. These morphological alterations were similar to those reported in studies with other compounds with anthelmintic properties [21,22]. Taken together, the in vitro schistosomicidal activities of amiodarone, telmisartan, and propafenone are in alignment with alterations in the tegumental surfaces of *S. mansoni*.

Similar to other antischistosomal agents, including the only schistosomicidal drug available worldwide (praziquantel), the mechanism by which these cardiovascular agents exert their effect on *S. mansoni* is still not clear. For example, amiodarone, a potassium channel blocker, affected the viability of all schistosomes during in vitro screening, but quinidine, which belongs to the same class, did not. Likewise, among angiotensin II receptor antagonists, only telmisartan had anthelmintic activity. Although we did not observe discernible schistosomicidal properties in many cardiovascular agents (listed in Table 1, but not shown in Figure 2), it does not necessarily mean those drugs do not have such an effect. For example, quinoline was found to exert in vivo antischistosomal properties [23], but these aminoquinoline compounds are known to exhibit anthelmintic action in vitro at high concentrations [9]. The reason we did not find apparent antischistosomal effects in our experiments for these drugs may be due to our use of relatively low concentrations of the drugs (primary screening at 50 µM), which might have reduced the chance of observing antischistosomal activity.

Other issues may be involved in the schistosomicidal effect of these cardiovascular drugs. Taking the antiarrhythmic drug amiodarone as an example, although classified as a potassium channel blocker, amiodarone interacts allosterically with muscarinic receptors [24] and it also blocks voltage-sensitive sodium channels [25]. As muscarinic receptors have been described in *S. mansoni,* and G protein-coupled receptors (GPCRs) are a potential candidate for antischistosomal drug targeting [26], the possibility of the action of amiodarone on the helminth’s muscarinic receptors cannot be excluded. In addition, various antiparasitic compounds, including praziquantel, target ion channels of the schistosome’s neuromuscular system [27,28]. In trypanosomatids such as *Trypanosoma cruzi* and *Leishmania mexicana,* it was found that amiodarone disrupts calcium homeostasis [29,30]. Thus, amiodarone’s effects may be mediated by perturbation of the ion channels. A similar reasoning can be applied to antiarrhythmic drug propafenone, a beta-adrenoceptor antagonist that also acts on the voltage-controlled ion channel [31].

Oral therapeutics are ideal for potential antischistosomal drug development. The five compounds active in vitro against *S. mansoni* fall into this category. Two of these drugs are anti-arrhythmic (amiodarone and propafenone) and three are anti-hypertensive (telmisartan, methyldopa, and doxazosin). However, not all have the potential for translational use in schistosomiasis therapy. The EC_50_ values of amiodarone fall into the therapeutic range (detected in serum levels) for their indicated therapies. Therapeutic serum levels are extremely variable from patient to patient, and effective treatments cover a concentration range, reaching up to 11.99 μg/mL (equivalent to 18.6 μM) [32].

Based on this evidence and considering the safety of amiodarone (the LD_50_ of oral amiodarone in mice exceeds 3000 mg/kg), we decided to evaluate the therapeutic action of amiodarone in mice infected with *S. mansoni*. Importantly, as praziquantel has a low efficacy on immature worms, we used a mouse model of schistosomiasis for both early and chronic *S. mansoni* infections. Interestingly, amiodarone treatment was more effective in early infection than in chronic infection. Although the worm and egg burden reductions achieved were only moderate, comparatively, treatment with amiodarone exceeded praziquantel in early infection (reduction of 52–60% for amiodarone vs. reduction ~23% for praziquantel). The egg burden reduction, mainly recorded in early *S. mansoni* infection, could be attributed to a decrease in the number of worms as a result of treatment with amiodarone and/or the reduction in egg-laying by female parasites. As schistosome parasites do not multiply in the mammalian host, these reductions in the number of worms and eggs are important because they are associated with a reduction in the morbidity of the disease. Possibly, a combined treatment with amiodarone and praziquantel might increase the therapeutic efficacy, and this topic may be explored in future studies. The use of amiodarone and praziquantel may also minimize the development of drug resistance by the schistosomes and open possibilities for further studies.

Clinically, the starting dosage of amiodarone is 800–1600 mg per day (taken by mouth in either a single dose or separate doses for 1–3 weeks), whereas the continuing dosage of amiodarone is 600–800 mg per day (taken by mouth in a single dose or separated doses for 1 month). Using a dose translation formula from humans to mice [33], a dose of 100 and 400 mg/kg in our mouse model of schistosomiasis is equivalent, respectively, to doses of 8 and 32 mg/kg in humans, in accordance with the clinical use of amiodarone (27 mg/kg/day). Thus, our data suggest that amiodarone should have antischistosomal properties under its current antiarrhythmic drug regimen. Although the amiodarone is commonly used in clinical practice, the risk of adverse effects acts as a limiting factor to its utilization [34,35,36,37,38,39,40,41,42,43,44,45,46,47]. As community-wide treatment would be expected at least in most endemic areas for schistosomiasis, this would be a problem. However, many of the safety issues reported in clinical studies with amiodarone are especially in the long term [47]. If effective against schistosomes, the use of amiodarone orally in a single dose or daily for 3–5 days may be advantageous.

## 4. Materials and Methods

### 4.1. Reagents and Drugs

All chemicals were of reagent grade quality or better, obtained from commercial suppliers and used without further purification. Cardiovascular drugs were purchased from Sigma-Aldrich (St. Louis, MO, USA), Cayman Chemical (Ann Arbor, MI, USA), and Toronto Research Chemicals (Toronto, ONT, Canada). Praziquantel was kindly provided by Ecovet Industria Veterinaria Ltda (Sao Paulo, Brazil). Roswell Park Memorial Institute (RPMI 1640) culture medium containing phenol red, and l-glutamine, penicillin G/streptomycin sulfate solution, and inactivated fetal bovine serum were obtained from Vitrocell (Campinas, Brazil). Dimethyl sulfoxide (DMSO), glutaraldehyde solution, and HEPES buffer were purchased from Sigma-Aldrich. In all in vitro experiments, drugs were solubilized in DMSO (0.5% in RMPI *v*/*v*), whereas for in vivo studies, drugs were dissolved in ethanol 2% in water (*v*/*v*).

### 4.2. Animals and Parasite Maintenance

A Brazilian strain of *S. mansoni* (Belo Horizonte, Brazil) was maintained by passage through mice and *Biomphalaria glabrata* snails, as definitive and intermediate hosts, respectively, at the Adolfo Lutz Institute (São Paulo, Brazil) [15]. Female Swiss mice weighing ~20 g each, 3 weeks old, were purchased from Animais para Laboratório (Anilab, São Paulo, Brazil). Both snails and mice were kept under environmentally controlled conditions (25 °C; humidity of 50%) and light cycles (12 h light and 12 h dark), with free access to water and food. For parasite maintenance, infected snails were induced with light to shed infectious larvae (cercariae) and mice were infected subcutaneously with 120 *S. mansoni* cercariae each. Rodents were randomly housed in individually vented caging systems in groups of five animals per cage.

### 4.3. Primary Screening

An in vitro primary screening against adult schistosomes was performed as previously described [35,36]. Briefly, adult parasites were collected from the portal system and mesenteric veins of infected mice 7 weeks post-infection (parasite ex vivo). Next, schistosomes were placed in RPMI 1640 culture medium supplemented with 10% fetal bovine serum, containing 100 IU/mL of penicillin and 100 μg/mL of streptomycin, and incubated in a 24-well culture plate (Corning, New York, NY, USA) at 37 °C and 5% CO_2_. Drugs were dissolved in DMSO to obtain stock solutions of 10 mM and were then tested at a concentration of 50 μM (one pair of parasites per well). Each drug was assessed in three replicates. Helminths were monitored microscopically and their viability was determined at 2, 24, 48, and 72 h [37].

### 4.4. Secondary Screening

The compounds that produced an effect greater than 80% after 72 h post-exposure in the primary screening were further tested using 1:2 serial dilutions from 0.78 to 50 µM for determination of their effective concentration 50% (EC_50_) and 90% (EC_90_) [13,38]. Each concentration was tested in five replicates, and experiments were repeated once. The negative control (using the highest concentration of DMSO, i.e., 0.5) and positive control (praziquantel 2 µM) were included.

### 4.5. Scanning Electron Microscopy Analysis

Scanning electron microscopy studies were performed as previously described [39,40]. Briefly, adult worms exposed to amiodarone, propafenone, and telmisartan or the DMSO vehicle for 24 h were fixed for at least 3 h in 2.5% glutaraldehyde at room temperature. After fixing, the samples were then dehydrated with increasing concentrations of ethanol at 50%, 70%, 90%, and 100%. Samples were then air-dried, mounted, and coated with gold sputter (Denton Vacuum LLC, Moorestown, NJ, USA) before imaging. Specimens were then observed and photographed using a high-resolution scanning electron microscope with an accelerating voltage of 20 kV (Jeol-JSM-6460LV, Tokyo, Japan).

### 4.6. In Vivo Studies in an Animal Model of Schistosomiasis

The compound that produced an EC_50_ < 10 μM during secondary screening was further tested in an animal model of schistosomiasis. For in vivo efficacy studies, mice were each infected subcutaneously with 80 *S. mansoni* cercariae. Animals were then randomly divided into experimental groups (five mice per group), and amiodarone was tested using a single oral dose (400 mg/kg) or once daily for 5 consecutive days (100 mg/kg/day) 21 days post-infection (immature stage, early infection) or 49 days post-infection (adult stage, chronic infection) [15,41]. For comparison, praziquantel was administered at 400 mg/kg to groups of five *S. mansoni*-infected animals in the same period. As a note, the doses used of amiodarone and praziquantel were based on the protocols recommended for experimental schistosomiasis [10,11,12,13,14]. For each treatment period, infected but only vehicle-treated mice (five mice per group) served as controls. On day 63 post-infection, animals in all groups were euthanized using CO_2_.

For determination of worm burden, schistosomes were collected by portal perfusion, and also collected from the mesenteric veins manually to ensure that all parasites had been collected [42]. Therapeutic efficacy was also based on the Kato–Katz method for quantitative examination of fecal eggs, and the number of eggs per gram was calculated [43]. For additional evaluation of the therapeutic efficacy, the percentages of different egg developmental stages (oogram pattern) were studied according to Pellegrino and colleagues [44], in which eggs at different stages of maturity were identified and the mean number of each stage was calculated. The percentage of worm and egg reduction was calculated by means of the following equation [45]:

% R = ([UC − TC]/UC) × 100%(1)

where:

R is the percentage of reduction;

UC is the mean of the untreated control group;

TC is the mean of the treated group.

### 4.7. Randomization and Blinding

Animal studies are reported in compliance with the National Centre for the Replacement, Refinement, and Reduction of Animals in Research (NC3Rs) ARRIVE guidelines. The animals were randomly assigned to their experimental groups, and they were also euthanized in a similarly random manner within their corresponding group in accordance with the standard operating procedures. All results obtained were analyzed by investigators blinded to the group conditions. Analyses were conducted by two different investigators according to standard procedures [10].

### 4.8. Statistical Analysis

Statistical analyses were performed using GraphPad Prism version 7 in accordance with the recommendations in the pharmacology field [10]. All data from the in vitro anthelmintic experiments are presented as the mean ± standard deviation (SD) of at least three independent assays. EC_50_ and EC_90_ values were calculated using sigmoid dose–response curves and 95% confidence intervals [46,47]. The overall survival of the adult schistosomes was determined by comparison using Kaplan–Meier survival curves and the log rank (Mantel–Cox) test [12]. For in vivo studies, a parametric Dunnett’s test was applied to compare the control group with the treated group. *p* values < 0.05 were considered statistically significant.

## 5. Conclusions

In the present work, from a phenotypic screening of 46 commercially available cardiovascular drugs, we found that the antiarrhythmic drug amiodarone exhibited antischistosomal properties with EC_50_ value < 10 μM, which is below the clinically achievable plasma concentrations in vivo. Furthermore, oral treatment with amiodarone was more effective than the gold-standard antiparasitic drug in mice harboring early *S. mansoni* infection. These results revealed the potential of amiodarone as a translatable lead for developing antischistosomal drugs. Furthermore, our findings also revealed the potential of telmisartan, propafenone, methyldopa, and doxazosin (EC_50_ values ranging from 12 to 38 µM) as antischistosomal molecules, and this opens possibilities for further studies in medicinal chemical optimization.

## Figures and Tables

**Table 1:** In vitro activity of cardiovascular agents against *S. mansoni* adult worms.

	Drug	Class	EC_50_ (µM)	EC_90_ (µM)
1	Atenolol	Beta-Adrenoceptor Antagonist	>50	>50
2	Bisoprolol		>50	>50
3	Carvedilol		>50	>50
4	Esmolol		>50	>50
5	Metoprolol		>50	>50
6	Nebivolol		>50	>50
7	Propranolol		>50	>50
8	Propafenone		25.7 ± 4.2	40.9 ± 3.5
9	Amlodipine	Calcium Channel Blocker	>50	>50
10	Diltiazem		>50	>50
11	Felodipine		>50	>50
12	Isradipine		>50	>50
13	Nifedipine		>50	>50
14	Nitrendipine		>50	>50
15	Verapamil		>50	>50
16	Amiodarone	Potassium Channel Blocker	8.2 ± 0.8	11.4 ± 1.2
17	Quinidine		>50	>50
18	Benazepril	Angiotensin-Converting Enzyme	>50	>50
19	Captopril	(ACE) Inhibitor	>50	>50
20	Enalapril		>50	>50
21	Fosinopril		>50	>50
22	Lisinopril		>50	>50
23	Perindopril		>50	>50
24	Quinacril		>50	>50
25	Ramipril		>50	>50
26	Losartan	Angiotensin II Receptor Antagonist	>50	>50
27	Olmesartan		>50	>50
28	Telmisartan		11.5 ± 1.6	20.6 ± 3.7
29	Valsartan		>50	>50
30	Doxazosin	Alpha-Adrenoceptor Antagonist	37.8 ± 3.3	49.4 ± 2.8
31	Prazosin		>50	>50
32	Clonidine	Alpha2-Adrenoceptor Agonist	>50	>50
33	Guanabenz		>50	>50
34	Methyldopa		30.1 ± 3.8	45.6 ± 4.3
35	Hydralazine	Vasodilator	>50	>50
36	Isosorbide mononitrate		>50	>50
37	Nitroglycerin		>50	>50
38	Nitroprusside		>50	>50
39	Papaverine		>50	>50
40	Propatilnitrate		>50	>50
41	Dobutamine	Beta1-Adrenergic Receptor Agonist	>50	>50
42	Etilefrine	Alpha1-Adrenoceptor Agonist	>50	>50
43	Digoxin	Cardiac Glycoside; Na^+^/K^+^-ATPase Inhibitor	>50	>50
44	Sildenafil	Phosphodiesterase Type 5 (PDE5) Inhibitor	>50	>50
45	Warfarin	Anticoagulant	>50	>50
46	Clopidogrel	Antiplatelet Aggregation	>50	>50
47	Praziquantel		0.98 ± 0.3	1.4 ± 0.2

EC_50_ and EC_90_ values were determined at 72 h. Values represent mean ± standard deviation of three independent experiments.

## Data Availability

Data is contained within the article.
